# The Interaction of *Helicobacter pylori* with TFF1 and Its Role in Mediating the Tropism of the Bacteria Within the Stomach

**DOI:** 10.3390/ijms20184400

**Published:** 2019-09-07

**Authors:** Marguerite Clyne, Felicity E. B. May

**Affiliations:** 1 School of Medicine and Conway Institute, University College Dublin, Belfield, 4, D04 V1W8 Dublin, Ireland; 2 Northern Institute for Cancer Research, Faculty of Medical Sciences, University of Newcastle upon Tyne, Newcastle-upon-Tyne NE2 4HH, UK

**Keywords:** *Helicobacter pylori*, TFF1, lectin, MUC5AC, lipopolysacchairde

## Abstract

*Helicobacter pylori* colonises the human stomach and has tropism for the gastric mucin, MUC5AC. The majority of organisms live in the adherent mucus layer within their preferred location, close to the epithelial surface where the pH is near neutral. Trefoil factor 1 (TFF1) is a small trefoil protein co-expressed with the gastric mucin MUC5AC in surface foveolar cells and co-secreted with MUC5AC into gastric mucus. *Helicobacter pylori* binds with greater avidity to TFF1 dimer, which is present in gastric mucus, than to TFF1 monomer. Binding of *H. pylori* to TFF1 is mediated by the core oligosaccharide subunit of *H. pylori* lipopolysaccharide at pH 5.0–6.0. Treatment of *H. pylori* lipopolysaccharide with mannosidase or glucosidase inhibits its interaction with TFF1. Both TFF1 and *H. pylori* have a propensity for binding to mucins with terminal non-reducing α- or β-linked N-acetyl-d-glucosamine or α-(2,3) linked sialic acid or Gal-3-SO_4_^2−^. These findings are strong evidence that TFF1 has carbohydrate-binding properties that may involve a conserved patch of aromatic hydrophobic residues on the surface of its trefoil domain. The pH-dependent lectin properties of TFF1 may serve to locate *H. pylori* deep in the gastric mucus layer close to the epithelium rather than at the epithelial surface. This restricted localisation could limit the interaction of *H. pylori* with epithelial cells and the subsequent host signalling events that promote inflammation.

## 1. Introduction

### 1.1. Site-Specific Tropism of Helicobacter Pylori in the Stomach

Approximately 60% of the global human population is infected with *Helicobacter pylori* which means that *H. pylori* is one of the commonest pathogens of mankind. The prevalence of infection ranges from 70% in Africa to approximately 35% in North America and Western Europe or 25% in Australia and New Zealand [1]. Infection with *H. pylori* clusters in families [2]. Individuals are normally infected in early life [3] and infection persists for the individual’s lifetime unless eradicated with antimicrobials. *Helicobacter pylori* colonises the gastric mucosa and induces a complex inflammatory response that causes chronic antral gastritis in both adults and children [4,5]. While the majority of those infected are asymptomatic, up to 10% develop duodenal ulceration. *Helicobacter pylori* is classed as a group I carcinogen because infection is associated with the development of gastric cancer [6,7] of which the vast majority of cases are of non-cardia gastric cancer [8].

One of the most striking characteristics of *H. pylori* is its species and tissue specificity. This microorganism only infects humans or non-human primates and the stomach is the only reservoir that *H. pylori* can be consistently isolated from. Although the organism can be found deep in the gastric glands [9,10] and a recent study in a murine model suggested that this *H. pylori* population may provide a long-term reservoir [11], the majority of bacteria are located in the gastric pits and in the mucus overlying the epithelial cells with only a small percentage attached to the epithelial cells [12,13]. *Helicobacter pylori* are found in the duodenum and oesophagus but only at sites of gastric metaplasia [14] which emphasises their specific tropism for the gastric mucosal surface. 

*Helicobacter pylori* has been shown to colocalise with the mucin MUC5AC that is secreted by normal gastric surface mucosal cells [15,16]. *Helicobacter* *pylori* does not adhere to intestinal metaplastic cells in the stomach that have a complete intestinal phenotype and express the mucin MUC2 but does adhere to metaplastic cells that have an incomplete intestinal phenotype and retain expression of MUC5AC [17,18]. Further, MUC5AC is expressed in the areas of gastric metaplasia in the duodenum and oesophagus that *H. pylori* can colonise [14]. The above findings provide strong evidence that MUC5AC, or a molecule that is co-expressed with MUC5AC, mediates the sequestration of *H. pylori* within the adherent gastric mucus gel layer.

An adherent mucus gel layer lines the stomach in its entirety [19]. This adherent mucus layer is thicker than adherent mucus layers elsewhere in the gastrointestinal tract, presumably because it is required to protect the gastric epithelium from the high hydrochloric acid concentration, low pH environment of the gastric lumen, and from digestion by pepsin. There is a hydrogen ion concentration gradient across the adherent mucus layer from 1.1 × 10^−4^ M, pH 6.96 at the junction between the gastric epithelium and the adherent mucus, to 5.6 × 10^−3^ M, pH 2.25 in the lumen [20]. Bicarbonate secretion into the mucus layer by the epithelium neutralises the hydrogen ions that diffuse from the gastric lumen into the mucus layer to generate the pH gradient [20]. 

The adherent mucus gel layer has been reported to have a multi-laminated structure formed by overlapping layers of mucins [13], MUC5AC, derived from the gastric surface mucus-secretory cells and MUC6 from the gland mucus-secretory cells. *Helicobacter* *pylori* colonises preferentially within the MUC5AC layer of mucin [13]. Despite causing chronic infection in the stomach, *H. pylori* is not an acidophile [21] and infection of experimental animals demonstrates that it colonises a narrow anatomical niche of the adherent mucus gel layer within 25 μm of the epithelium [22], at which the pH is near neutral. *Helicobacter* *pylori* has been reported to interact with MUC5AC via outer membrane protein adhesins and O-linked oligosaccharides displayed on the mucin [23,24,25,26].

### 1.2. Site-Specific Localisation of Trefoil Proteins and Co-Expression with Mucins in the Gastrointestinal Tract

The trefoil factor family proteins, TFF1, TFF2 and TFF3, are small secreted proteins thought to play a role in mucosal protection and repair and to help maintain mucus integrity possibly by cross-linking mucins to aid in the formation of stable mucus gel layers [27,28,29]. Trefoil proteins contain trefoil domains that are characterised by several conserved amino acid residues. Six cysteine residues, with conserved spacing form three inter-domain disulphide bonds with common cysteine pairing [27,29]. Both TFF1 and TFF3 have a single trefoil domain and an extra-trefoil domain seventh cysteine three residues from each of their carboxy-termini that is available to form inter-molecular covalent bonds [29,30,31]. TFF2 contains two trefoil domains and two extra-trefoil domain cysteine residues that form a disulphide bond between its amino and carboxy termini [29]. TFF1, TFF2 and TFF3 were identified originally as an oestrogen-responsive gene product in human breast cancer cells [32,33], a constituent of porcine pancreatic protein preparations [34] and a rat intestinal protein [35], respectively. It is recognised now that TFF1, TFF2 and TFF3 are synthesised and secreted predominantly by normal secretory epithelia [27,36]. 

TFF3 is expressed most widely, followed by TFF1 with expression of TFF2 being more restricted [27,36]. For example, TFF1 and TFF3 are present in the respiratory system with strong immunoreaction for TFF3 in the goblet cells of bronchi [37]. Both proteins are also expressed in normal breast epithelial cells [38,39]. TFF1 and TFF3 are detected in the male and female reproductive tracts with changes to endometrial TFF3 expression coincident with the implantation window. Expressed frequently alongside mucins, the idea evolved that individual trefoil factors are more likely to be co-expressed with individual mucins. It is in the gastrointestinal tract that trefoil factor expression and co-localisation with mucins has been studied most, especially in hollow organs though their expression in the endocrine pancreas is recognised. Both TFF1 and TFF3 are produced in the minor salivary glands in the mouth and in the submucosal glands in the oesophagus [40]. TFF1 and TFF2 are expressed in the stomach and all three trefoil proteins are expressed in the duodenum [41]. TFF1 is detected in Brunner’s glands of the duodenum and in some goblet cells, TFF2 is expressed in large amounts exclusively in Brunner’s glands of the duodenum and TFF3 in large amounts exclusively in all goblet cells of the duodenum. Thereafter, in the jejunum, ileum, colon and rectum, expression of TFF3 predominates; it is present in all goblet cells alongside the secreted mucin, MUC2, whereas TFF1 is expressed in only a subset of goblet cells. As well as being secreted into the gastrointestinal lumen, TFF1 is synthesised in gastric and intestinal, and TFF3 in intestinal, enteroendocrine or neuroendocrine cells for secretion into the lamina propria to exert paracrine actions or thence, after release into the circulation, for transport through the vasculature to exert endocrine actions [40].

**Figure 1:**
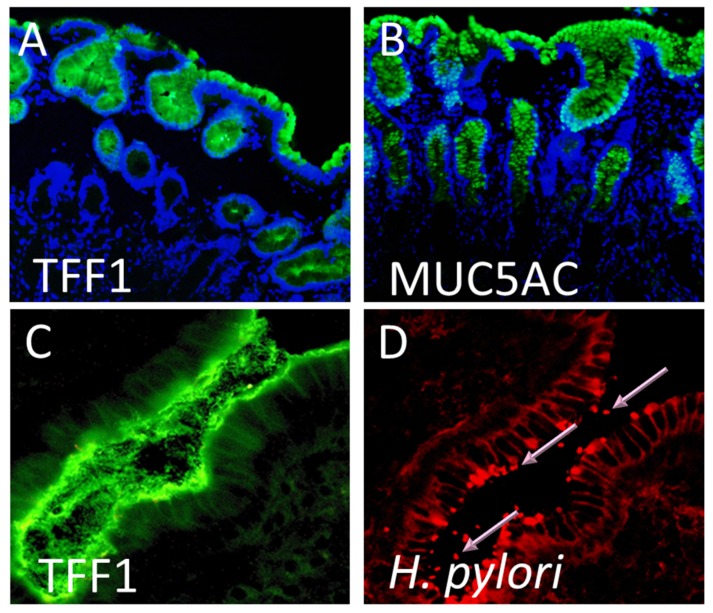
Trefoil factor 1 (TFF1), the mucin MUC5AC, and *Helicobacter* *pylori* are found at the same sites in the human stomach. Formalin-fixed gastric mucosal tissue from *H. pylori*-negative individuals were immunofluorescently stained using specific antibodies against (**A**) TFF1 and (**B**) MUC5AC. Both TFF1 (green) and MUC5AC (green) staining occurred on gastric surface foveolar cells and in the glands. Cell nuclei were counter stained with DAPI (blue). Original magnification 100×. A frozen section of *H. pylori* infected antral gastric biopsy tissue was immunofluorescently stained using specific antibodies against (**C**) TFF1 and (**D**) *H. pylori*. Both TFF1 (green) and *H. pylori* (red) were detected at the epithelial surface and in the overlying gastric mucus. Pink arrows indicate *H. pylori* organisms staining bright red. The same field is shown in (**C**,**D**). Original magnification 200×.

Within the stomach, TFF1 is expressed in large amounts in the mucus secretory foveolar cells throughout the stomach and is detected packaged alongside the secreted mucin, MUC5AC, in mucin granules of the apical mucous secretory vesicles ready for release into the adherent mucus layer [42] (Figure 1A,B). The membrane mucin, MUC1 is co-expressed also by the gastric surface mucous secretory cells. TFF2 is detected in the mucus neck cells of the gastric fundus and body in the basal glands in the antrum and pylorus [41,43]. It co-localises within these cells with the secreted mucin, MUC6. TFF3 is not expressed by normal human stomach [40,44].

The adherent mucus gel layer was destroyed by conventional fixation prior to histology or immunohistochemistry. Examination of carefully preserved cryosections allowed us to demonstrate high concentrations of TFF1 throughout the adherent mucus layer with apparent concentration in the lower half of the mucus layer next to the epithelial cells [45]. Thus, the close association between TFF1 and MUC5AC detected in mucus granules of foveolar cells [42] was maintained following secretion into the adherent mucus layer. However, although TFF1 was present throughout the mucus layer, it was concentrated at the higher-pH end of the pH gradient proximal to the epithelial surface.

Three molecular forms of TFF1 were detected in normal gastric mucosa and adherent mucus, a 6.67 kDa monomer, a 13.33 kDa homodimer and an ~25 kDa heterodimer [45] with another secreted protein, the 18.3 kDa TFIZ1 [46] encoded by *GKN2* [47]. Early analysis by caesium chloride density gradient centrifugation demonstrated that the TFF1 homodimer, and to a lesser extent the monomer, banded with mucin glycoproteins [45]. The association between TFF1 homodimer and mucins resisted disruption with guanidine hydrochloride consistent with the interaction being non-ionic, possibly hydrophobic in nature. Analysis under physiological conditions demonstrated that the endogenous gastric TFF1 homodimer is associated with MUC5AC but not MUC6 because it co-elutes with the former but not the latter after fractionation by native gel filtration [42]. This association between TFF1 dimer and MUC5AC was confirmed by immunoprecipitation with antibodies against each protein followed by non-reducing western transfer analysis and detection with antibodies against the other. An involvement of divalent cations in the interaction between MUC5AC and TFF1 homodimer was indicated by disruption of the interaction by incubation with EGTA and confirmed by the increased association obtained in the presence of Ca^2+^. 

The co-expression of TFF1 with the gastric mucin MUC5AC and the co-localisation of *H. pylori* with both TFF1 and MUC5AC (Figure 1) led us to hypothesise that an interaction between *H. pylori* and TFF1 could explain the distinct tropism that the organism has for the gastric mucosa. 

## 2. *Helicobacter* *pylori* Interacts with the Dimeric Form of TFF1

To test the above hypothesis, we investigated if there is an interaction between *H. pylori* and TFF1. Co-localisation of *H. pylori* and TFF1 was shown by immunofluorescence in parallel sections of gastric biopsies from *H. pylori*-infected individuals [48]. A direct interaction between *H. pylori* and recombinant TFF1 dimer coated onto latex beads was demonstrated in a flow cytometric adherence assay. Five *H. pylori* strains, two strains of *Campylobacter jejuni* and *Escherichia coli* stain HB101 were tested. All five strains of *H. pylori* bound to TFF1-coated beads but no binding was detected for the two *C. jejuni* strains or for *E. coli*. Binding of *H. pylori* to the beads was inhibited by pre-incubation of the TFF1 dimer-coated beads with a TFF1 monoclonal antibody [48]. Furthermore, the interaction of *H. pylori* with beads coated with the monomeric form of TFF1 was negligible. 

The interaction of *H. pylori* with TFF1 dimer was confirmed by surface plasmon resonance (SPR) [48], which allows real-time monitoring of molecular interactions at a sensor surface [49]. We used a CM5 sensor chip which is a glass slide with a thin layer of gold on one side covered with a covalently bound carboxymethylated (CM) dextran matrix attached by a hydroxyalkyl thiol linker layer. Light energy generated when there is an interaction with the gold film generates SPR and changes resulting from binding interactions between ligands and proteins immobilized on the chip can be monitored. We immobilised TFF1 to the sensor chip and allowed *H. pylori* to be passed over the surface. *Helicobacter* *pylori* interacted specifically with TFF1 dimer bound to the sensor chip and not with an uncoated dextran chip. The IC_50_ for inhibition of *H. pylori* binding to TFF1 dimer by TFF1 in solution was 30.5 ng/mL and 1.56 µg/mL TFF1 abolished binding completely which demonstrates that the *H. pylori* interaction was with TFF1. An early study reported that TFF1 and TFF3 could be detected on the surface of *Staphylococcus aureus* organisms present in the lumen of infected airways and in sputum from cystic fibrosis patients based upon co-localisation of immunofluorescence [37], but *H. pylori* is the only bacterium that has been shown to interact physically with a member of the trefoil factor family of proteins. The identification of TFF1 as a binding partner for *H. pylori* might play a role in enabling this organism to persist in gastric mucus and cause chronic infection. It also opens up the possibility that TFF1 or TFF1 analogues might be used to either treat or prevent infection with *H. pylori*. 

## 3. The Interaction of *H. pylori* with TFF1 is Mediated via Glycans Present on the Core Oligosaccharide Region of Lipopolysaccharide

**Figure 2:**
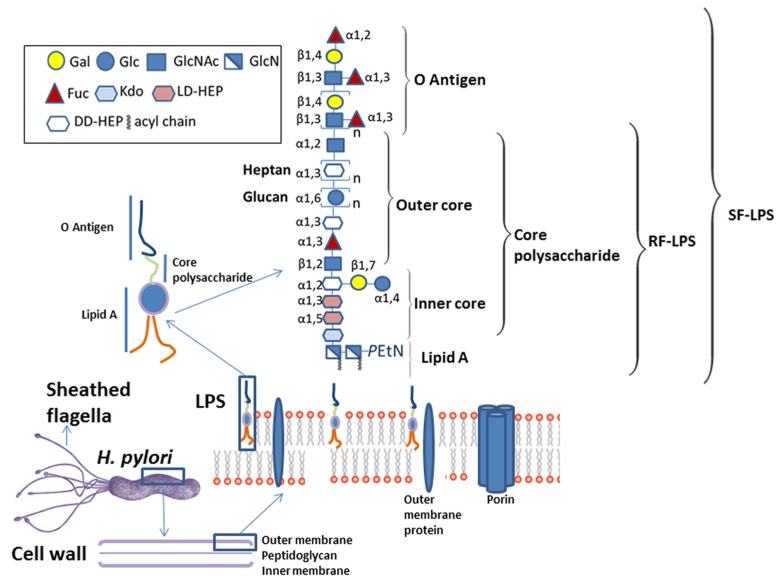
Components of the surface of *H. pylori* that have the potential to interact with host molecules include the flagella, outer membrane proteins and lipopolysaccharide (LPS). The location of *H. pylori* flagella, the structure of the Gram-negative bacterial cell wall together with the detailed composition of *H. pylori* LPS as described by Li et al [59] are shown.

The demonstration that *H. pylori* interact specifically with TFF1 raised the question of how this interaction might occur. *Helicobacter* *pylori* is a 3 µm long helix-shaped rod-like bacterium of 0.5 µm diameter. Its motility is conferred by 4 to 6 sheathed monopolar flagella composed of two flagellin subunits, FLaA and FLaB [50]. The flagellar sheath is a continuation of the bacterial phospholipid outer membrane and is thought to protect the flagella filament from gastric acid. The outer-membrane contains, as do all Gram-negative bacteria, lipopolysacharide (LPS) (Figure 2). *Helicobacter* *pylori* LPS comprises lipid A, which anchors it in the phospholipid outer membrane, a core oligosaccharide, which together with lipid A maintains the integrity of the outer membrane and the O-antigen, characterised by fucosylated and nonfucosylated N-acetyllactosamine saccharide units connected to the core and extending on the outer surface of the bacteria [51,52,53,54]. Lipid A of *H. pylori* has a unique structure; it is less acylated and less phosphorylated than lipid A from other bacteria which reduces its endotoxic activity [55] and increases its resistance to cationic microbial peptides [56]. The O-antigen can mimic the host Lewis antigens [53] which allows *H. pylori* to evade immune recognition [57]. Lipopolysaccharide that contains lipid A and the inner and outer core oligosaccharides are generally referred to as rough-form LPS (RF-LPS), whereas the complete LPS capped with the O-antigen is called smooth-form LPS (SF-LPS) (Figure 2). A number of outer membrane proteins of *H. pylori* have been identified as adhesins [23,25,26] as has the Lewis × (Le^x^) blood group epitope that is part of the LPS O-antigen [58]. The outer membrane of *H. pylori* might plausibly interact with TFF1 via one or more of these bacterial cell surface molecules.

The specificity of possible interactions was investigated initially by SPR [60]. The TFF1 dimer inhibited binding of *H. pylori* to a TFF1-coated dextran chip; TFF3 dimer was ten-fold less potent and TFF2 was unable to displace the bacterium. Thus, interaction of *H. pylori* is specific for the TFF1 dimer. TFF1 dimer but not TFF1 monomer interacted with an ~6 kDa molecule from *H. pylori* lysates after their separation by denaturing polyacrylamide gel electrophoresis and transfer to polyvinylidene difluoride (PVDF) membrane. Subsequent analysis of *H. pylori* fractions demonstrated that the ~6 kDa molecule is present in the bacterium’s outer membrane and flagella but not in its inner membrane. This molecule interacted with Alcian blue which stains glycosylated molecules consistent with the molecule containing glycan. Pre-incubation of *H. pylori* lysates with proteinase K did not affect interaction of TFF1 with the ~6 kDa molecule nor its detection with Alcian blue but pre-incubation with sodium metaperiodate prevented the interaction with TFF1 and detection with Alcian blue [60], consistent with the interaction being between TFF1 dimer and a *H. pylori* saccharide. These data strongly suggested that LPS on the surface of *H. pylori* mediates the interaction of *H. pylori* with TFF1. 

Alcian blue detects also a prominent ~6 kDa molecule corresponding to RF-LPS and less well two larger molecules shown to contain O-antigens in purified *H. pylori* LPS after separation by denaturing gel electrophoresis. Recombinant TFF1 dimer interacted with purified *H. pylori* RF-LPS, specifically with the ~6 kDa molecule, but not with higher molecular mass SF-LPS after electrophoresis and transfer to PVDF membrane. An interaction was confirmed by altered non-denaturing gel electrophoretic mobility of TFF1 after incubation with RF-LPS and by detection of binding of TFF1 dimer to an *H. pylori* RF-LPS-coated chip by SPR. The interaction was inhibited by pre-incubation of *H. pylori* RF-LPS with mannosidase and glucosidase whereas incubation with galactosidase and fucosidase was ineffective. Pre-incubation of TFF1 with mannose and glucose together but not with either alone reduced the interaction between TFF1 dimer and *H. pylori* RF-LPS demonstrating that the interaction involves probably both mannose and glucose [60]. 

An early study of the core oligosaccharide of *H. pylori* indicated that it contains glucose and mannose [61]. A more recent study compared LPS of two well-characterised *H. pylori* strains [62], the weakly inflammatory, mouse-adapted strain SS1 [63] and the potent inflammatory *H. pylori* Type I strain G27 [64]. LPS was extracted by the hot-phenol water method and the aqueous and phenol layers analysed by gas chromatography–mass spectrometry. Significant amounts of xylose, mannose, galactose, glucose, heptose, and N-acetylglucosamine were detected in LPS fractions from both strains but the abundance of the different monosaccharides varied between the strains and their fractions; mannose was more abundant in strain G27 than in strain SS1 [62]. It was suggested that the failure of other studies to show the presence of mannose in *H. pylori* LPS [50] is due to the differences in the culture conditions of the bacteria [55].

Comparison of the interactions of TFF1 and *H. pylori* with purified samples of animal and human mucins [65] detected a remarkable association between the affinities of TFF1 and *H. pylori* for different gastric mucin preparations (Spearman’s rho = 0.81; *p* = 0.002). Characterisation of the accessible saccharide lectin affinities in the mucin preparations favoured by TFF1 indicated that there was a preferential interaction with terminal non-reducing α- or β-linked N-acetyl-d-glucosamine or α-(2,3)-linked sialic acid or Gal-3-SO_4_^2−^. If a direct interaction with these saccharides, which were not tested in the earlier study [60], was to be demonstrated, it would establish that TFF1 is able to interact with a small subset of saccharides. 

**Figure 3:**
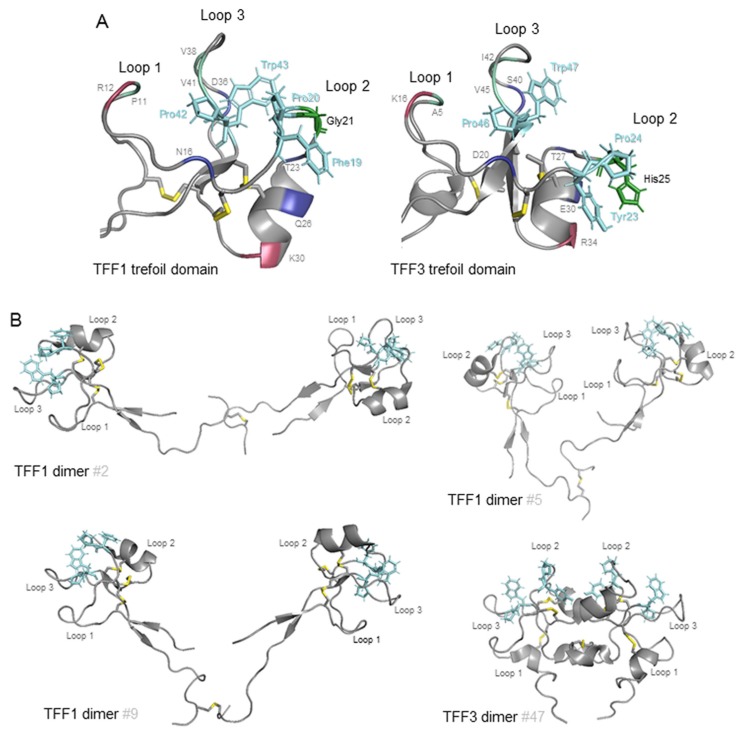
Hydrophobic surfaces of TFF1 and TFF3. Representations of the backbones of the TFF1 and TFF3 trefoil domains [57,60] (**A**) and TFF1 and TFF3 dimers [58,59] (**B**) are shown. The intramolecular and intermolecular disulphide bonds are shown and coloured yellow. The three fully conserved solvent accessible residues, Pro 20, Pro42 and Trp43 in TFF1, and one semi-conserved solvent accessible residue, Phe19 in TFF1, in loops 2 and 3 that form a continuous hydrophobic patch postulated to interact with a saccharide or aromatic amino acid sidechain between loops 2 and 3 are shown in stick representation and are coloured cyan (**A**,**B**). The backbones of the surface residues with conserved features that surround this patch are indicated in single letter code (**A**). Of these, the hydrophobic residues, Pro11 (P11), Val38 (V38) and Val41 (V41) in TFF1, are coloured pale green, the hydrogen bond donor, Arg12 (R12) is coloured raspberry red and the hydrogen bond acceptors, Asn16 (N16), Thr23 (T23), Gln26 (Q26) and Asp36 (D36) in TFF1, are coloured deep blue. Two other well-conserved residues on the edge of the hydrophobic patch are indicated. A positively charged residue, Lys30 (K30) in TFF1, is coloured raspberry red. The small Gly21 in TFF1 that would facilitate access to the hydrophobic cleft and the corresponding His25 residue of TFF3 proposed to impede access are shown in stick representation and are coloured forest green (**A**). Three of the solution NMR structures of the TFF1 dimer, #2, #5 and #9 and one of the TFF3 dimers (#47), are shown with the first monomer unit on the left-hand side.

The demonstration that TFF1 interacts with specific carbohydrates of *H. pylori* RF-LPS begs the question, with which part of TFF1 do *H. pylori* interact? TFF1 is an exceptionally highly charged small acidic protein with a pI of 3.94 [31]. It has, however, a region between the second and third loops of its mature, folded trefoil domain (Figure 3A) that consists largely of solvent-accessible, hydrophobic residues and includes the aromatic residues Phe19, Pro20, Pro42, and Trp43 [66]. It has been suggested that this small hydrophobic patch, which has a cleft-like shape and is present in all trefoil domains, is a potential binding pocket for an oligosaccharide or forms part of a protein binding site that includes hydrophobic interaction with side-chains of aromatic residues such as proline or tryptophan. Comparison of the structures of the hydrophobic clefts present in the four human trefoil domains, confirms that conserved residues either form the core of the domain or are located around the cleft [29]. Formed mainly from the peptide backbone, the only side chains that contribute are those of the absolutely conserved Pro20, Pro42 and Trp43 in TFF1 and the semi-conserved Phe19 of TFF1 that is replaced by a tyrosine in TFF2’s second trefoil domain and in TFF3 [29,66]. Several residues that flank these aromatic amino acid residues and have conserved properties provide additional interactions that could confer specificity to interaction with a trefoil factor ligand or receptor (Figure 3A). The presence of Gly21 on the edge of the TFF1 hydrophobic cleft, and glycine residues in the equivalent positions in both TFF2 trefoil domains, is likely to facilitate access of any ligand whereas the presence of His25 in the equivalent positon in TFF3 is likely to impede access. Notably, a further difference among these potential binding pockets in TFF1 and TFF3 is that, whereas in TFF1 the cleft is narrow, 6 Å, in TFF3 it is much wider, 11–12 Å, and has a more shelf-like structure. Should this groove prove to be the site of interaction of TFF1 with *H. pylori* RF-LPS, it is perhaps not surprising that although the TFF3 molecule is able to interact, its affinity is reduced because its binding pocket has incomplete sequence conservation with and is much larger than the TFF1 binding pocket [29,67]. 

The TFF1 dimer that interacts preferentially with *H. pylori* RF-LPS has two of these small hydrophobic patches one on each monomer unit [68]. The higher affinity of the TFF1 dimer than the TFF1 monomer for *H. pylori* may indicate that the same binding surface from each monomer unit is involved in the interaction with *H. pylori*. Alternatively, different surfaces may be required and the spatial accessibility or physical proximity is provided best by two covalently bound units. The two trefoil domains are connected by a flexible region that comprises the amino- and carboxy-termini of each monomeric unit [68]. The distance between the α carbons of the Pro20 residues of the putative binding surface varied between 36 and 73 Å and the spatial orientations of the two monomer units were divergent in the different molecular structures; the two trefoil domains did not adopt a fixed orientation with respect to each other (Figure 3B). In the structure on the top left, #2, the TFF1 monomer units were in the same orientation with both hydrophobic surfaces on the same face of the molecule orientated towards the viewer. In the structure on the top right, #5, the TFF1 monomer units are much closer together and are in opposite orientations with the two loop 1s proximate and with the first hydrophobic surface orientated away from the viewer and the second towards the viewer. In the structure on the bottom left, #9, the monomer units are again in opposite orientations but with the two loop 2s proximate and with the first hydrophobic surface orientated towards the viewer and the second away from the viewer. In contrast, in the TFF3 dimer structure, #47, the monomer units were a relatively fixed distance apart of 13–19 Å between the α carbons of the Pro24 residues, and were always in the same opposite orientation with the two loop 2s proximate and with both hydrophobic surfaces on the same face of the molecule but facing in opposite directions. Thus, the distance between and spatial orientations of the monomer units in the TFF1 dimer have inherent flexibility and adaptability, at least between pH 5.6 and 7.6, that is not provided by the TFF3 dimer [67,68]. This versatility might facilitate crosslinking of *H. pylori* to other molecules such as mucins. 

## 4. The Interaction of *H. pylori* with TFF1 Explains Site-Specific Localisation of the Bacteria Within the Stomach

**Figure 4:**
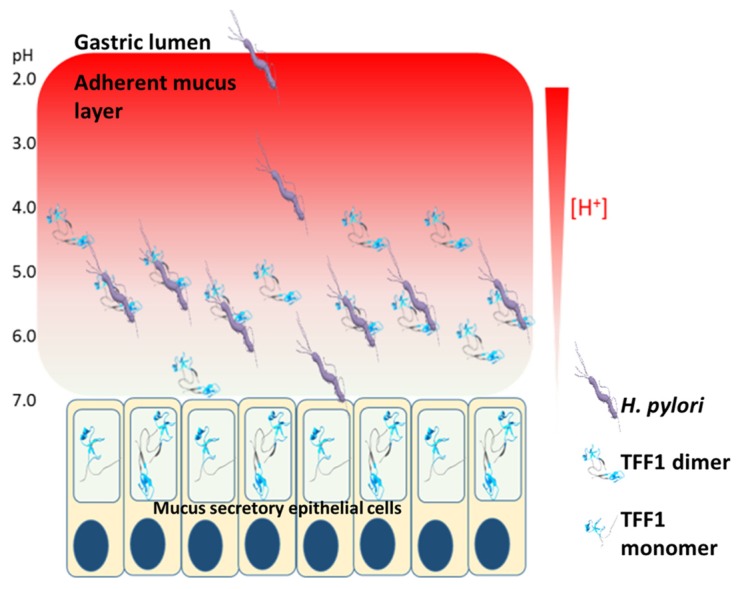
Potential biological effect of *H. pylori* interaction with TFF1. TFF1 is localised within mucous granules in mucus-secreting cells and is secreted from the cells into the adherent mucus layer. TFF1 is concentrated close to the epithelial surface at the higher pH end of the pH gradient. Upon entering the stomach, *H. pylori* travel through the mucus layer to escape the acidic environment present in the gastric lumen. *H. pylori* reside in a niche close to the gastric epithelium where the pH is near neutral. Binding of *H. pylori* to TFF1 occurs best at pH 5.0–6.0 and may anchor *H. pylori* deep in the mucus layer close to the epithelial cells. This interaction may limit the number of organisms that reach and interact with the epithelial cells, thus reducing the inflammatory response.

The unique adaptation of *H. pylori* to the harsh acidic environment of the stomach is facilitated by its expression of a urease that assists neutralisation of its microenvironment [21]. However, *H. pylori* are not localised predominantly in the gastric lumen nor are they found in significant concentrations in association with gastric mucosal surface epithelial cells. Rather, *H. pylori* colonise the adherent mucus gel layer. They burrow, with the assistance of their flagella into the mucus and down into a preferred layer by chemotaxis stimulated by avoidance of high H^+^ concentrations [22,69]. The specific tropism of *H. pylori* for the inner layer of the adherent mucus gel layer led us to investigate the pH dependence of the TFF1 dimer interaction with *H. pylori* RF-LPS. The interaction in solution was significantly greater at pH 5.0 and 6.0 than at either pH 4.0 or 7.0. The pH preference at pH 5.0 and 6.0 was investigated by analysis of the interaction of TFF1 dimer with fractionated and immobilised *H. pylori* RF-LPS; interaction was slightly higher at pH 6.0 than pH 5.0. *Helicobacter* *pylori* colonise a region of the 100 µm thick adherent mucus gel layer 0–25 µm above the epithelium in an animal model [22]. The optimum pH for the interaction between TFF1 dimer and *H. pylori* RF-LPS suggests that this interaction may facilitate the localisation of *H. pylori* in the adherent mucus gel layer a little above the junction between the epithelial layer and the mucus layer (Figure 4). The optimum binding for TFF3 homodimer to RF-LPS was pH 7.0 and was considerably less than that observed for TFF1 at pH 5.0–6.0 [60]. It is plausible that *H. pylori* could bind to TFF3 in the intestine at pH 7.0. However, this interaction would likely occur at the intestinal lumen which would result in removal of the bacteria due to mucus turnover. Such an interaction could be one of the reasons why we do not see *H. pylori* in the intestine. 

We assessed if *H. pylori* binding to TFF1 could promote mucus colonisation [70]. The HT29-MTX-E12 cell line is a subclone of the colorectal adenocarcinoma cell line HT29-MTX, that forms tight junctions and produces an adherent mucus layer when grown on Transwell filters [71]. Production of the secreted mucin MUC5AC and cell surface expression of the membrane-bound mucin MUC1 was demonstrated by immunofluorescence. A few cells produced the intestinal mucin MUC2. The cells produced all three human trefoil factors, but the immunoreaction for TFF2 was weaker than for TFF1 and TFF3. The extracellular adherent mucus gel layer contained MUC5AC, TFF1 and TFF3 [70]. *Helicobacter* *pylori* infected the cells and formed discrete clusters within the mucus layer in close association with TFF1. An isogenic mutant of *H. pylori* with a truncated LPS core colonised the HT29-MTX-E12 mucus layer less well than the wild-type parent strain indicating that the full length core-oligosacchairde is required for interaction with TFF1. Furthermore, pre-incubation of cells with LPS from the wild-type strain but not with LPS from the mutant strain reduced colonization [70]. These results demonstrate that the interaction of the core oligosaccharide of *H. pylori* LPS with TFF1 can promote colonisation of mucus. Interestingly, commensal *E. coli* strains engineered to produce recombinant fimbriae that express fusion trefoil factor proteins at their tips bound more mucin than *E. coli* transfected with an empty vector [72], which is further evidence that TFF1 present on the surface of bacteria can promote binding to mucin. 

Analysis of different clinical isolates found that there was considerable diversity in both the level of glycosylation and the length of the fucosylated O-antigen chains among strains isolated from either the same or different individuals [73]. Of note, reduced Lewis antigen expression was found when isolates were grown at pH 5.0 compared to growth at pH 7.0 [73]. Reduced presence of SF-LPs would expose more RF-LPS on the bacterial surface and would enhance the interaction of *H. pylori* with TFF1. We hypothesise that the interaction of *H. pylori* with TFF1 that is favoured at pH 5.0–6.0 serves to locate *H. pylori* in the gastric mucus layer close to the epithelium rather than at the epithelial surface. This restricted localisation could limit the number of *H. pylori* that interact with epithelial cells and, thus, reduce host signalling events that cause inflammation (Figure 4). In support of this hypothesis, it has been shown that *H. pylori* strains isolated from asymptomatic, infected individuals tend to express lower molecular mass RF-LPS than those isolated from individuals with severe gastroduodenal disease [73,74,75]. In addition, *H. pylori* infection of TFF1-null mice causes more severe gastric inflammation than infection of wild-type mice [76], which suggests that an increased number of organisms may interact with the epithelial cells if TFF1 is absent from the gastric mucus than if it is present.
